# Gastrobronchial Fistula after Robotic Repair of Traumatic Diaphragmatic Hernia

**DOI:** 10.1155/2020/8085425

**Published:** 2020-03-15

**Authors:** Jamie T. Tung, Leah M. Lucero, James W. Davis, Lawrence P. Sue

**Affiliations:** UCSF Fresno Department of Surgery, Community Regional Medical Center, 2823 Fresno Street, Fresno, CA 93721, USA

## Abstract

Gastrobronchial fistulas are a rare occurrence in the literature. We report a case of a gastrobronchial fistula after robotic repair of a chronic traumatic diaphragmatic hernia. The patient had severe respiratory symptoms with multiple studies that were inconclusive. The fistula was ultimately discovered after an esophagogastroduodenoscopy (EGD). The patient underwent a left thoracotomy for takedown of his fistula and eventually recovered. Earlier EGD and a lower threshold for differential that included this diagnosis would have led to an earlier identification and treatment of a rare disease process.

## 1. Introduction

Fistulous communications between the stomach and lung are rare. All previous cases have been only described as case reports which makes their study difficult given their rarity and sparse descriptions in the literature [1–4]. We report the only known case of a gastrobronchial fistula following a robotic diaphragm repair. Our patient illustrates the diagnostic challenge and biases that may come with such a rare case.

## 2. Case Presentation

A 45-year-old man was ejected in a motor vehicle collision and taken to Community Regional Medical Center, the region's Level I trauma center. He was hemodynamically normal on arrival. His history included a previous motor vehicle collision approximately 20 years prior, at which time he underwent a left thoracotomy and splenectomy. The patient was unable to recall any other details of injuries or surgical interventions from this prior accident. Examination was significant for a left thoracotomy scar. Computed tomography (CT) scan revealed a large left-sided diaphragmatic hernia. His other injuries were relatively minor and did not require operative intervention. The diaphragmatic hernia was suspected to be chronic given the lack of any significant trauma, symptoms, and the history of a suspected previous repair. Given the likely chronic nature of the hernia, the decision was made to perform the repair semielectively.

On hospital day 2, the patient was taken for a robotic-assisted, laparoscopic repair of his diaphragmatic hernia. An abdominal approach was chosen due to surgeon discretion, as the patient had both previous abdominal and thoracic surgeries and thus adhesions were likely to be encountered with either approach. At exploration, the hernia was found to be lateral and posterior, and a significant amount of scar tissue was dissected further confirming its chronic nature. The diaphragm was unable to be completely primarily repaired, and a Phasix™ ST Mesh was used as an interposition graft with Prolene sutures to secure it to the diaphragm.

Initially, the patient did well; however, on postoperative day 5, he had an episode of desaturation and a large amount of hemoptysis. A CT was done and showed significant intra-abdominal and mediastinal air with the suspicion of tracheobronchial injury. The patient was intubated and a bronchoscopy was performed, which revealed blood in the left bronchus but no discrete lesion. A repeat bronchoscopy the following day showed large amounts of clot and mucous casts from the left lung, but again, no discrete defect.

While in the ICU, he had multiple episodes of bilious emesis that filled his ventilator circuit. The first episode occurred approximately two weeks following his index operation. He had a worsening chest X-ray and P/F ratio, suggestive of ARDS. Multiple modes of ventilation were tried to improve his PaCO_2_. In an effort to decrease the bilious emesis, his endotracheal tube was changed and nasogastric tube replaced.

**Figure 1:**
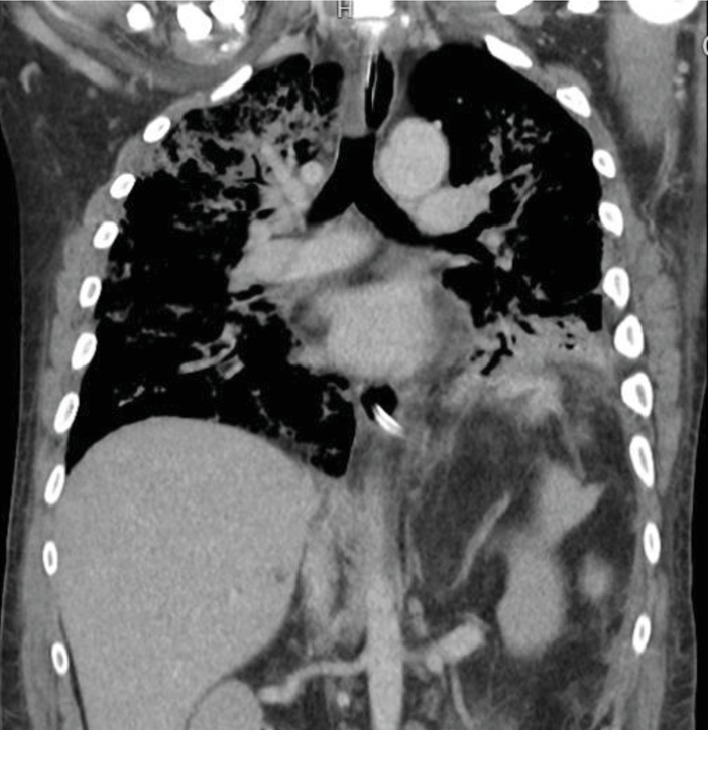
CT scan of left lower lobe lung injury secondary to the gastrobronchial fistula and oral contrast within the lung.

As he continued to have repeated episodes of bilious emesis filling his ventilator circuit, a diagnosis of a gastrobronchial fistula was entertained. For further workup, an abdominal CT scan with oral contrast was ordered and demonstrated contrast in the left lower lobe (Figure 1). This was attributed to aspiration by the radiologist. The pulmonology service was consulted for repeat bronchoscopy to evaluate for possible gastrobronchial fistula. They declined as they had never heard of the diagnosis and felt that the trauma service should be investigating more likely causes. An upper gastrointestinal (GI) series was negative. An esophagogastroduodenoscopy (EGD) was performed by the primary team and clearly showed two areas with air blowing into the stomach, in synchrony with the ventilator, along the greater curvature and visible PDS suture in the gastric lumen.

The patient was taken to the operating room for a left thoracotomy. The gastrobronchial fistula was identified. It appeared that the previous diaphragm repair sutures had incorporated the diaphragm and the stomach but did not involve the lung tissue. As the left lower lobe was found to be unsalvageable, a left lower lobectomy, wedge resection of stomach, and diaphragm repair were performed. Eventually, the patient's condition improved, and he was able to have a tracheostomy performed and weaned from the ventilator.

## 3. Discussion

Gastrobronchial fistulas are very rare in the reported literature. A related but distinct entity of gastropleural fistulas also exists, when the fistula enters the thoracic cavity but does not communicate with a bronchus. These two related entities likely arise from similar mechanisms and are only reported as case studies. The literature is varied in all aspects of these fistulas from their cause, mechanism, method of diagnosis, and treatment. The time course to diagnosis and development varies from within days to several years of initial insult [1–4].

While the mechanism of these fistulas is not known for certain, the most prevalent theory describes an inciting event that causes erosion between the stomach and lung tissue. If this erosion only invades into the pleural space without invading the lung tissue, it would likely cause a gastropleural fistula. Abscesses, trauma, hernias, malignancies, and postsurgical complications have all been reported to cause these fistulas. Abscesses and malignancies are easily understood mechanisms, as erosion into surrounding structures is a hallmark of these disease processes. Traumatic injuries presumably result in fistula formation at the time of insult. Hernias likely cause these fistulas due to repeated sliding of the stomach creating an ulcer that allows for erosion into the lung tissue [1, 5]. Lastly, postsurgical changes from dissection or staples can cause ischemic changes that create an environment for fistula formation [6–8].

No case of a gastrobronchial fistula has resulted as a complication of a repair of a traumatic diaphragmatic hernia. Given that suture material was visualized on EGD, the most likely explanation is that the initial insult leading to fistula formation was from a technical error with some of the stomach incorporated into the diaphragm repair suture. Another factor that could have contributed to the fistula formation is the error in using the Phasix™ ST Mesh to bridge the diaphragm gap as it is not designed to be used in such a manner. This could have led to the failure of the repair allowing the stomach to herniate through the diaphragm against the lung. In addition, the uncoated side of the mesh would have been against the lung tissue, a possible contributing factor to the fistula formation.

This is the only case study that describes fistula formation occurring after a robotic-assisted laparoscopic repair of a chronic diaphragmatic hernia, as most gastrobronchial fistulas from an operation have been described after laparoscopic sleeve gastrectomies [9].

Clinical presentation is highly variable—particularly timing of onset. Some patients develop symptoms immediately postoperatively while others present in a delayed fashion. Severity of symptoms varies from mild to severe, such as ARDS secondary to exposure to caustic gastric contents. Some authors have found presenting symptoms to point to an obvious diagnosis while others have been delayed by incongruent diagnostic data, as in our case. Due to the variability of clinical presentations, diagnosis is challenging and must include a high index of suspicion. At present, the diagnosis has revolved around three modalities: barium swallow (fluoroscopy or CT), bronchoscopy, and EGD [1, 5, 6, 10, 11].

We encountered significant diagnostic bias given that none of the providers from multiple services had previously encountered this entity. Additionally, multiple nondiagnostic or negative studies contributed. In retrospect, the upper GI study was positive and potentially diagnostic but incorrectly interpreted. Other authors have found that the results may be impacted by patient positioning [5, 11].

Successful management has been described with nonoperative treatment with gastric aspiration and nutrition, minimal intervention with endoscopic treatment, and surgical via abdominal laparotomy or thoracotomy [5–8, 12, 13]. The treatment selected must be individualized to the particular clinical picture, respiratory status, and overall health and stability of the patient. In our case, the patient had a continually worsening status, and surgical intervention was the only treatment option that would allow him to survive.

In retrospect, a more expeditious diagnosis would have led to faster treatment. This case serves to highlight the importance of an open mindset with minimization of biases in approaching problems of noncongruent information.
